# Recent advances in central cardiovascular control: sex, ROS, gas and inflammation

**DOI:** 10.12688/f1000research.7987.1

**Published:** 2016-03-31

**Authors:** Pauline M. Smith, Alastair V. Ferguson

**Affiliations:** 1 Department of Biomedical and Molecular Sciences, Queen's University, Kingston, Ontario, K7L3N6, Canada

**Keywords:** hypertension, hydrogen sulfide, reactive oxygen species, reproductive hormones, cardiovascular homeostasis

## Abstract

The central nervous system (CNS) in concert with the heart and vasculature is essential to maintaining cardiovascular (CV) homeostasis. In recent years, our understanding of CNS control of blood pressure regulation (and dysregulation leading to hypertension) has evolved substantially to include (i) the actions of signaling molecules that are not classically viewed as CV signaling molecules, some of which exert effects at CNS targets in a non-traditional manner, and (ii) CNS locations not traditionally viewed as central autonomic cardiovascular centers. This review summarizes recent work implicating immune signals and reproductive hormones, as well as gasotransmitters and reactive oxygen species in the pathogenesis of hypertension at traditional CV control centers. Additionally, recent work implicating non-conventional CNS structures in CV regulation is discussed.

## Introduction

According to the World Health Organization (WHO), cardiovascular (CV) disease accounts for approximately 17 million deaths a year worldwide ^1^, of which more than half (9.4 million) are attributable to complications of hypertension ^2^. In 2008, a staggering 40% of adults over the age of 25 had been diagnosed with hypertension ^3^.

The central nervous system (CNS) is essential to maintaining CV homeostasis. Traditional central autonomic CV control centers include the nucleus tractus solitarius (NTS), the rostral ventral lateral medulla (RVLM), and the caudal ventral lateral medulla in the brainstem; the parabrachial nucleus in the pons; and the paraventricular nucleus (PVN) in the hypothalamus. In addition, the area postrema (AP) in the hindbrain, and the organum vasculosum of the lamina terminalis (OVLT) and subfornical organ (SFO) in the forebrain, are sensory circumventricular organs (CVOs) characterized by the presence of a wide variety of receptors and the lack of the normal blood-brain barrier, which have also been implicated in central CV regulation. The renin-angiotensin aldosterone system (RAAS) has also been extensively implicated as a critical signaling system, components of which play central roles both as circulating hormones and as CNS neurotransmitters in the regulation of blood pressure (BP). There is growing evidence that the development and progression of hypertension involves dysregulation of the sympathetic nervous system (SNS) (SNS over-activity) (for review, see 4– 6) and activation of the RAAS ^7, 8^.

Over the past 20 years, our understanding of CNS control of BP regulation (and dysregulation leading to hypertension) has evolved substantially. This review will summarize some of these paradigm shifts, focussing primarily on signaling molecules that either (i) are not classically viewed as CV signaling molecules (i.e. immune signals and reproductive hormones) or (ii) exert effects at CNS targets in a non-traditional manner, acting via membrane receptor-independent signaling mechanisms (i.e. gasotransmitters and reactive oxygen species [ROS]), all of which have been shown to have profound effects on the central control of BP. CNS structures, not conventionally thought of as CV control centers but that more recently have been shown to influence CV regulation, are also discussed.

## Inflammation and immune regulators as modulators of cardiovascular regulation and contributors to hypertension

Although it had been speculated decades ago that there was a relationship between the immune system and hypertension ^9^, the demonstration of systemic markers of inflammation in patients with essential hypertension in the early 2000s ^10, 11^ was a catalyst for renewed interest in the relationship between hypertension and the immune system. Emerging evidence suggests that both the innate and acquired immune systems are activated in hypertension, as inflammations in the kidney, vasculature (arteries), and CNS have all been shown to be involved in the pathogenesis of hypertension.

As an immediate first-line defence mechanism to infections or tissue injury, the innate immune system initiates a generalized inflammatory response involving dendritic cells, macrophages, natural killer (NK) T cells, and Toll-like receptors (TLRs), all of which have been shown to be activated in hypertension.

Dendritic cell activation has been shown to promote hypertension by stimulating T-cell proliferation which infiltrates both the kidney and arterial walls ^12, 13^. Similarly, macrophage infiltration of the kidney and arteries has been documented in experimental models of hypertension, and a decrease in macrophage infiltration is associated with an improvement of hypertension in these models of hypertension ^14– 17^. Recently, NK T-cell activation and TLRs (TLR4, in particular) have been suggested to play a role in hypertension-related inflammation ^18, 19^.

The adaptive immune system responds to specific antigens and involves antigen presentation, lymphocyte activation, and antibody production. T cells have been shown to play a role in angiotensin II (ANG II)-induced hypertension ^20^ whereas endogenously produced ANG II increases T-cell activation ^21^. Pro-inflammatory T-cell activation and the subsequent release of pro-inflammatory cytokines are associated with hypertension ^22– 24^ whereas inhibition or genetic ablation of the B7/CD28 T cell costimulatory pathway has been shown to prevent experimental hypertension ^12^. RAG-1 ^−/−^ mice and SCID mice, which lack both T and B cells, exhibited a blunted hypertensive response to ANG II infusion ^20, 25^, a response that returned when T cells were transferred into RAG-1 ^−/−^ mice ^20^. T cell-produced cytokines (such as tumor necrosis factor alpha, or TNFα) and many of the interleukins (such as IL-6) have been shown to play a role in hypertension. TNFα antagonism ^20, 26^ or genetic knockout of IL-6 ^27^ has been shown to blunt ANG II-induced hypertension. The presence of agonist antibodies to ANG II receptors has been identified in a number of conditions that are characterized by elevated BP, such as preeclampsia ^28, 29^, refractory hypertension ^30, 31^, and malignant hypertension ^32^.

Many studies have suggested that arterial inflammation within specific CNS locations is involved in the pathogenesis of hypertension. A role for inflammation in the NTS, a pivotal region for regulating arterial pressure baroreceptor reflex sensitivity, has been suggested in the development of hypertension, as studies have shown not only leukocyte accumulation within the NTS microvasculature ^33^ but also changes in gene expression of a variety of inflammatory molecules ^34, 35^ and neurotrophic factors ^36^ in the NTS of spontaneously hypertensive rats (SHRs).

In addition, many of the cytokines, released as a consequence of immune system activation, have been shown to directly influence cardiovascular control centers in the CNS. Microinjection of IL-6 into the NTS attenuates baroreceptor function ^37^ and leads to speculation that abnormal gene expression of IL-6 in the NTS may be associated with hypertension. Augmentation of IL-1β, IL-6, or TNF-α expression and increased ROS observed in the RVLM following chronic intraperitoneal lipopolysaccharide administration have been suggested to be contributing factors to neurogenic hypertension induced by systemic inflammation ^38^.

Early studies identified the anteroventral third ventricle (AV3V), a broad-based region located along the wall of the third ventricle which includes the OVLT, as a critical CNS structure in the pathogenesis of hypertension ^39^. A more recent study not only confirmed that lesions of the AV3V region attenuate ANG II-induced hypertension but also implicated immune system involvement as AV3V lesions eliminated circulating T-cell activation and vascular infiltration normally observed in response to ANG II administration ^40^. IL-1β has been shown to influence the excitability of SFO neurons ^41^, and recent studies have demonstrated that microinjection of IL-1β (and of TNFα) into SFO increases BP and renal sympathetic nerve activity (SNA) ^42^.

The PVN, a hypothalamic autonomic control center with well-documented roles in CV regulation, has been implicated as a CNS structure in which immune signals may act to cause hypertension. Chronic ANG II infusion causes the expression of pro-inflammatory cytokines and markers of oxidative stress in the PVN, effects blocked by central administration of TNFα blocker ^26^. Angiotensin-converting enzyme 2 (ACE2) overexpression in the PVN has also been shown to attenuate both ANG II-induced hypertension and expression of the pro-inflammatory cytokines TNFα, IL-1β, and IL-6 in the PVN ^43^. Blockade of nuclear factor-kappa-B (NFκB), a prominent transcription factor that governs inflammatory responses, in the PVN of rats resulted in decreased BP, pro-inflammatory cytokines, and ROS, as well as upregulation of key protective anti-hypertensive RAAS components, suggesting an important role for NFκB in PVN in the hypertensive response ^44^. Finally, rats fed a high-salt diet demonstrated increased expression of IL-1β and decreased expression of the anti-inflammatory cytokine IL-10, in the PVN. These expression levels were augmented by stimulation of ROS production within the PVN ^45^.

## Reproductive hormones and cardiovascular regulation

The interest in the role of sex hormones in hypertension has been driven by a number of observations regarding sexual dimorphism in BP regulation in humans and animals. Epidemiological findings that prior to menopause the prevalence of essential hypertension is lower in women than in men of the same age ^46^ and that young women have lower resting SNA than men ^47^, differences that disappear after menopause, suggest that estradiol is important in BP regulation and, in fact, may protect against hypertension. Findings that estradiol administration attenuates increases in BP normally exhibited by intact males and ovariectomized females, and prevents development of hypertension in experimental models of hypertension ^48, 49^, suggest a role for estradiol in the regulation of BP.

Studies in humans and animals suggest that exogenous testosterone may also play a crucial role in BP regulation. In humans, low testosterone levels have been correlated with higher BP ^50, 51^ whereas testosterone replacement has been shown to cause significant reductions in BP ^52, 53^, suggesting a role for testosterone in BP regulation. Moreover, in experimental models of hypertension high BP develops more rapidly and becomes more severe in the male than in the female, effects which were shown to be androgen-dependent ^48, 54, 55^. Further support for a role of testosterone in the etiology of hypertension is derived from studies showing that castration prevents the development of hypertension in SHR rats ^56^.

Evidence for a role for central actions of estradiol on BP regulation is derived from a variety of sources. Firstly, many of the CNS sites with well-documented roles in CV regulation have been shown to possess estrogen receptors (ERα and ERβ) ^57– 61^. Moreover, intracerebroventicular (icv) administration of estradiol in ovariectomized mice and in male mice attenuated the increase in BP normally elicited by ANG II ^62^. In rats, aldosterone/salt-induced hypertension is exhibited by intact males and ovariectomized females, effects attenuated by activation of central ER receptors. Central ER blockade ^63^ or icv injections of small interfering RNA-ERα (siRNA-ERα) or siRNA-ERβ ^64^, on the other hand, augmented aldosterone-induced hypertension in intact females.

Further to these findings, estradiol has been shown to act via ERα or ERβ (or both) at specific brain regions in both males and females to influence sympathetic outflow and baroreflex function. The AP and SFO predominantly express ERα ^57– 62^, and estradiol has been shown to decrease the activity of AP ^65^ and SFO neurons ^66^, and inhibits ANG II activation of AP ^67^ and SFO neurons ^66^, whereas genetic knockdown of ERα in the SFO enhances ANG II-induced hypertension in female mice ^68^.

Estrogen actions at ERβ in PVN inhibit hypertensive effects of glutamate activation ^69^. In the RVLM, estradiol actions at ERβ receptors have been shown to cause decreases in BP in normotensive rats ^70^ and to attenuate aldosterone-induced increases in SNA and BP ^64^ whereas ERβ knockdown in RVLM or PVN results in the augmentation of aldosterone-induced increases in SNA and BP ^64^, effects that are not seen in intact females ^64^.

Relaxin, a member of the insulin family best known for its role in pregnancy, has also been shown to influence BP. Early studies revealed that chronic intravenous (iv) administration of relaxin elicited a decrease in BP in SHRs ^71^. Relaxin binding sites and relaxin receptors have been shown to be widely distributed throughout the brain, including the SFO, NTS, and PVN ^72^, suggesting that relaxin may be involved in the central control of BP. Hypertensive effects of central administration of relaxin into the dorsal third ventricle are totally abolished by lesions of the SFO ^73^, identifying this CVO as one central target mediating these cardiovascular effects. A recent study demonstrating that acute microinjection of relaxin-2 into the PVN increased sympathetic outflow and BP in SHR, whereas chronic PVN administration caused a profound increase in BP in normotensive rats ^74^, supports the conclusion that there are multiple central targets for this reproductive hormone/neurotransmitter. Moreover, this same study revealed that neutralization of endogenous relaxin reduced BP in SHR but had no significant effect in WKY ^74^, suggesting a role for relaxin in the pathogenesis of hypertension.

Another reproductive peptide that warrants further investigation into its potential contribution to the pathogenesis of hypertension is prolactin, a hormone best known for its involvement in lactation and reproduction. Very few studies have investigated the role of prolactin in the central control of CV regulation despite epidemiological evidence suggesting correlations between circulating prolactin levels and increased BP. Plasma prolactin has been shown to be elevated in patients with essential hypertension ^75^ and preeclampsia ^76, 77^. Furthermore, higher plasma prolactin levels have been shown to be associated with increased risk of hypertension in menopausal ^78^ and post-menopausal ^79^ women and in preeclampsia ^54^. Prolactin receptors are widely distributed throughout the body ^80^ and brain ^81^. mRNA for the prolactin receptor has been reported in the PVN ^81, 82^, and we have identified the presence of the prolactin receptor at levels similar to the AT1 receptor in the SFO ^83^. However, to our knowledge, studies investigating the CV consequences of central administration of prolactin (icv or microinjection into discrete brain nuclei) on BP, or the effects of prolactin on neuronal excitability in central CV control centers, are lacking.

## Gasotransmitters and cardiovascular regulation: hydrogen sulfide

Gasotransmitters are endogenously produced membrane permeable gas molecules which act at specific, targeted cells via membrane receptor-independent signaling mechanisms to exert well-defined physiological effects. The action(s) of nitric oxide (NO) and carbon monoxide (CO) at peripheral tissues and in the CNS to influence cardiovascular regulation are well documented ^84, 85^. More recently, a third gasotransmitter, hydrogen sulfide (H _2_S), an environmental air pollutant with well-known deleterious health effects, has been identified and suggested to play a role in the pathogenesis of hypertension. H _2_S is endogenously produced from catalysis of L-cysteine by using four enzymes: cystathionine β-synthase (CBS), cystathionine γ-lysase (CSE), or 3-mercaptopyruvate sulfur transferase (3MST) in tandem with cysteine aminotransferase (CAT). CBS is highly expressed in the CNS where it produces H _2_S from L-cysteine ^86^, whereas CSE is the predominant enzyme expressed in the myocardium and vasculature smooth muscle cells ^87^. Though predominantly found in the mitochondria where they work in tandem to produce H _2_S, 3MST and CAT are also expressed in the brain and vascular endothelium ^88^. In addition, H _2_S can be produced in red blood cells by the conversion of polysulfides which are obtained from dietary sources ^89^.

Evidence for a role of H _2_S in the pathogenesis of hypertension is suggested by the observation that plasma H _2_S concentrations are lower in patients with grade 2 or grade 3 hypertension, portal hypertension, and pulmonary hypertension ^90– 92^ and in preeclampsia where plasma H _2_S levels and placental CBS mRNA expression are decreased ^93, 94^.

H _2_S has been shown to be endogenously produced in peripheral vascular tissues and has been demonstrated to be a potent vasodilator, causing vasorelaxation in mesenteric arteries ^95^, aortic rings ^96, 97^, the ductus arteriosis ^96^, and pulmonary arteries ^98^ via actions on vascular smooth muscle cells. Unlike its gasotransmitter counterparts, NO and CO, vascular smooth muscle relaxation occurs independently of cGMP pathway activation. Activations of Ca ^2+^-activated potassium channels (BKCa) ^99^, ATP-sensitive potassium channels (K _ATP_) ^100^, Kv7 voltage-gated potassium channels ^97^, and cytochrome P-450 2C (Cyp2C) ^99^ have all been implicated as mechanisms of the H _2_S vasorelaxation.

A bolus iv injection of H _2_S elicited an immediate depressor response in normotensive rats ^100^ whereas chronic intraperitoneal administration of H _2_S decreases BP in hypertensive rats ^101– 104^. These findings, along with the fact that mice lacking CSE exhibit hypertension and reduced endothelium-dependent vasorelaxation ^105^, provide evidence of a direct role for H _2_S in BP regulation.

A role for H _2_S in the central control of BP stems from studies demonstrating that icv administration of H _2_S has been shown to dose-dependently decrease BP, effects which are followed by potent long-lasting hypertension actions attributed to modulation of H _2_S on K _ATP_ channels and α adrenergic stimulation, respectively ^106^. Furthermore, microinjection of H _2_S into discrete brain nuclei known for their involvement in CV regulation has also been shown to affect BP. H _2_S administration into the RVLM elicits decreases in BP, effects again mediated by K _ATP_ channels ^107^, whereas similar microinjections into the PVN ^108^ and SFO ^109^ have been shown to dose-dependently increase BP. Moreover, H _2_S has been shown to influence the excitability of neurons in the NTS ^110^, PVN ^111^, and SFO ^109^, CNS areas involved in CV regulation.

## Reactive oxygen species and cardiovascular control

When produced at appropriate concentrations, ROS have been implicated in the regulation of many critical physiological processes, including cell signaling, maintenance of appropriate vascular tone, inflammation, and immune responses. ROS overproduction, on the other hand, is a feature common to a number of pathological conditions, including hypertension.

A role for ROS in hypertension is suggested in humans as a positive correlation between BP and biomarkers of oxidative stress in patients with essential hypertension has been reported ^112, 113^. Furthermore, mice lacking nicotinamide adenine dinucleotide phosphate (NADPH) oxidases, key enzymes in the production of ROS, are protected against experimental hypertension ^114^, whereas overexpression potentiates ANG II-induced hypertension ^115^.

ROS production in specific CNS cardiovascular control centers, including both brain stem (NTS, RVLM) and hypothalamic (PVN) nuclei, and within the CVOs (SFO) has been shown to play a role in neurogenic hypertension ^116– 118^. Superoxide dismutase (SOD), an enzyme that metabolizes superoxide, overexpression in the brain abolished the hypertensive response normally observed in response to icv ANG II administration ^119^, whereas specific SOD3 deletion in the SFO increased baseline BP and potentiated ANG II-induced increases in BP ^120^. Interestingly, this same study showed that ROS in the SFO leads to infiltration by activated lymphocytes in the peripheral vasculature ^120^, linking oxidative stress in the CNS with immune activation in the periphery, which in concert would serve to intensify hypertension.

A high-salt diet increases NADPH oxidase (NOX-2 and NOX-4) expression in the PVN, whereas microinjection of amino-triazole (ATZ), a catalase inhibitor which increases ROS, into the PVN augments renovascular hypertension as well as increasing BP in normal rats ^45^.

## A role for ‘other’ central nervous system structures in the central control of blood pressure

This review has focussed on actions of non-traditional CV signaling molecules at CNS structures with well-documented roles in CV regulation. Another emerging area that warrants mention is the role of CNS regions not classically viewed as CV control centers that have been suggested to play a role in the pathogenesis of hypertension, secondarily or as a co-morbidity to other disease states. For example, the explosion of obesity research further to the discovery of leptin in the 1990s ^121^ has highlighted the involvement of a number of CNS autonomic control centers not typically viewed as CV control centers, such as the arcuate nucleus and the anterior hypothalamus, in the pathogenesis of hypertension as a consequence of direct actions of metabolic signals in these areas (for review, see 122, 123). Furthermore, many metabolic signals associated with obesity have been demonstrated to influence BP regulation via actions at the ‘classical’ CNS CV control centers. Further study of the actions of traditional CV signals (such as ANG II) within these non-traditional CV CNS centers may elucidate previously unknown roles of these regions in normal CV regulation.

## Conclusions

In this brief review, we have highlighted some emerging new perspectives which over the past 20 years contributed new and important information to the evolution of our understanding of CNS mechanisms involved in central CV control. The areas we have chosen to discuss are far from an exhaustive list of what is new and interesting, but do emphasize that this is a continually developing area of research with an inherent complexity associated with the requirement for integration of diverse autonomic systems. This points us in the direction of understanding that we perhaps should not expect to consider either single brain areas or single signalling molecules as “cardiovascular” at the expense of also describing their roles in other systems. Such conclusions point us to the broader perspective that all of these brain areas, signaling molecules, and autonomic systems contribute to the complex homeostatic regulation which maintains our “milieu interior” in a state of optimal health.

## References

[1] World Health Organization: Causes of Death 2008 Reference Source

[2] Lim SS Vos T Flaxman AD: A comparative risk assessment of burden of disease and injury attributable to 67 risk factors and risk factor clusters in 21 regions, 1990-2010: a systematic analysis for the Global Burden of Disease Study 2010. *Lancet.* 2012;380(9859):2224–60. 10.1016/S0140-6736(12)61766-8 23245609 PMC4156511

[3] World Health Organization. Global status report on noncommunicable diseases 2011.

[4] DiBona GF: Sympathetic nervous system and hypertension. *Hypertension.* 2013;61(3):556–60. 10.1161/HYPERTENSIONAHA.111.00633 23357181

[5] Guyenet PG: The sympathetic control of blood pressure. *Nat Rev Neurosci.* 2006;7(5):335–46. 10.1038/nrn1902 16760914

[6] Parati G Esler M: The human sympathetic nervous system: its relevance in hypertension and heart failure. *Eur Heart J.* 2012;33(9):1058–66. 10.1093/eurheartj/ehs041 22507981

[7] Leenen FH: Actions of circulating angiotensin II and aldosterone in the brain contributing to hypertension. *Am J Hypertens.* 2014;27(8):1024–32. 10.1093/ajh/hpu066 24742639

[8] Moon JY: Recent Update of Renin-angiotensin-aldosterone System in the Pathogenesis of Hypertension. *Electrolyte Blood Press.* 2013;11(2):41–5. 10.5049/EBP.2013.11.2.41 24627703 PMC3950224

[9] White FN Grollman A: Autoimmune Factors Associated With Infarction Of The Kidney. *Nephron.* 1964;1(2):93–102. 10.1159/000179322 14248727

[10] Chae CU Lee RT Rifai N: Blood pressure and inflammation in apparently healthy men. *Hypertension.* 2001;38(3):399–403. 10.1161/01.HYP.38.3.399 11566912

[11] Engström G Janzon L Berglund G: Blood pressure increase and incidence of hypertension in relation to inflammation-sensitive plasma proteins. *Arterioscler Thromb Vasc Biol.* 2002;22(12):2054–8. 10.1161/01.ATV.0000041842.43905.F3 12482834

[12] Vinh A Chen W Blinder Y: Inhibition and genetic ablation of the B7/CD28 T-cell costimulation axis prevents experimental hypertension. *Circulation.* 2010;122(24):2529–37. 10.1161/CIRCULATIONAHA.109.930446 21126972 PMC3064430

[13] Xiao L Kirabo A Wu J: Renal Denervation Prevents Immune Cell Activation and Renal Inflammation in Angiotensin II-Induced Hypertension. *Circ Res.* 2015;117(6):547–57. 10.1161/CIRCRESAHA.115.306010 26156232 PMC4629828

[14] Boesen EI Williams DL Pollock JS: Immunosuppression with mycophenolate mofetil attenuates the development of hypertension and albuminuria in deoxycorticosterone acetate-salt hypertensive rats. *Clin Exp Pharmacol Physiol.* 2010;37(10):1016–22. 10.1111/j.1440-1681.2010.05428.x 20626757 PMC3612979

[15] Muller DN Shagdarsuren E Park JK: Immunosuppressive treatment protects against angiotensin II-induced renal damage. *Am J Pathol.* 2002;161(5):1679–93. 10.1016/S0002-9440(10)64445-8 12414515 PMC1850776

[16] Rodríguez-Iturbe B Pons H Quiroz Y: Mycophenolate mofetil prevents salt-sensitive hypertension resulting from angiotensin II exposure. *Kidney Int.* 2001;59(6):2222–32. 10.1046/j.1523-1755.2001.00737.x 11380825

[17] Rodríguez-Iturbe B Quiroz Y Nava M: Reduction of renal immune cell infiltration results in blood pressure control in genetically hypertensive rats. *Am J Physiol Renal Physiol.* 2002;282(2):F191–201. 10.1152/ajprenal.0197.2001 11788432

[18] Bomfim GF Dos Santos RA Oliveira MA: Toll-like receptor 4 contributes to blood pressure regulation and vascular contraction in spontaneously hypertensive rats. *Clin Sci (Lond).* 2012;122(11):535–43. 10.1042/CS20110523 22233532 PMC4004345

[19] Sollinger D Eißler R Lorenz S: Damage-associated molecular pattern activated Toll-like receptor 4 signalling modulates blood pressure in L-NAME-induced hypertension. *Cardiovasc Res.* 2014;101(3):464–72. 10.1093/cvr/cvt265 24302630

[20] Guzik TJ Hoch NE Brown KA: Role of the T cell in the genesis of angiotensin II induced hypertension and vascular dysfunction. *J Exp Med.* 2007;204(10):2449–60. 10.1084/jem.20070657 17875676 PMC2118469

[21] Hoch NE Guzik TJ Chen W: Regulation of T-cell function by endogenously produced angiotensin II. *Am J Physiol Regul Integr Comp Physiol.* 2009;296(2):R208–16. 10.1152/ajpregu.90521.2008 19073907 PMC2643984

[22] Bautista LE Vera LM Arenas IA: Independent association between inflammatory markers (C-reactive protein, interleukin-6, and TNF-alpha) and essential hypertension. *J Hum Hypertens.* 2005;19(2):149–54. 10.1038/sj.jhh.1001785 15361891

[23] Chamarthi B Williams GH Ricchiuti V: Inflammation and hypertension: the interplay of interleukin-6, dietary sodium, and the renin-angiotensin system in humans. *Am J Hypertens.* 2011;24(10):1143–8. 10.1038/ajh.2011.113 21716327 PMC3807212

[24] Madhur MS Lob HE McCann LA: Interleukin 17 promotes angiotensin II-induced hypertension and vascular dysfunction. *Hypertension.* 2010;55(2):500–7. 10.1161/HYPERTENSIONAHA.109.145094 20038749 PMC2819301

[25] Crowley SD Song Y Lin EE: Lymphocyte responses exacerbate angiotensin II-dependent hypertension. *Am J Physiol Regul Integr Comp Physiol.* 2010;298(4):R1089–97. 10.1152/ajpregu.00373.2009 20147609 PMC4422347

[26] Sriramula S Cardinale JP Francis J: Inhibition of TNF in the brain reverses alterations in RAS components and attenuates angiotensin II-induced hypertension. *PLoS One.* 2013;8(5):e63847. 10.1371/journal.pone.0063847 23691105 PMC3655013

[27] Lee DL Sturgis LC Labazi H: Angiotensin II hypertension is attenuated in interleukin-6 knockout mice. *Am J Physiol Heart Circ Physiol.* 2006;290(3):H935–40. 10.1152/ajpheart.00708.2005 16284237

[28] Siddiqui AH Irani RA Zhang W: Angiotensin receptor agonistic autoantibody-mediated soluble fms-like tyrosine kinase-1 induction contributes to impaired adrenal vasculature and decreased aldosterone production in preeclampsia. *Hypertension.* 2013;61(2):472–9. 10.1161/HYPERTENSIONAHA.111.00157 23283357 PMC4131732

[29] Wallukat G Homuth V Fischer T: Patients with preeclampsia develop agonistic autoantibodies against the angiotensin AT1 receptor. *J Clin Invest.* 1999;103(7):945–52. 10.1172/JCI4106 10194466 PMC408252

[30] Liao Y Wei YM Wang M: Autoantibodies against AT1-receptor and alpha1-adrenergic receptor in patients with hypertension. *Hypertens Res.* 2002;25(4):641–6. 10.1291/hypres.25.641 12358154

[31] Zhu F Sun YX Liao YH: Agonistic AT ^1^ receptor autoantibody increases in serum of patients with refractory hypertension and improves Ca ^2+^ mobilization in cultured rat vascular smooth muscle cells. *Cell Mol Immunol.* 2008;5(3):209–17. 10.1038/cmi.2008.26 18582403 PMC4651272

[32] Dragun D Müller DN Bräsen JH: Angiotensin II type 1-receptor activating antibodies in renal-allograft rejection. *N Engl J Med.* 2005;352(6):558–69. 10.1056/NEJMoa035717 15703421

[33] Waki H Hendy EB Hindmarch CC: Excessive leukotriene B4 in nucleus tractus solitarii is prohypertensive in spontaneously hypertensive rats. *Hypertension.* 2013;61(1):194–201. 10.1161/HYPERTENSIONAHA.112.192252 23172924 PMC4506640

[34] Gouraud SS Waki H Bhuiyan ME: Down-regulation of chemokine Ccl5 gene expression in the NTS of SHR may be pro-hypertensive. *J Hypertens.* 2011;29(4):732–40. 10.1097/HJH.0b013e328344224d 21358418

[35] Waki H Gouraud SS Maeda M: Contributions of vascular inflammation in the brainstem for neurogenic hypertension. *Respir Physiol Neurobiol.* 2011;178(3):422–8. 10.1016/j.resp.2011.05.004 21601658

[36] Gouraud SS Takagishi M Kohsaka A: Altered neurotrophic factors' expression profiles in the nucleus of the solitary tract of spontaneously hypertensive rats. *Acta Physiol (Oxf).* 2016;216(3):346–57. 10.1111/apha.12618 26485190

[37] Takagishi M Waki H Bhuiyan ME: IL-6 microinjected in the nucleus tractus solitarii attenuates cardiac baroreceptor reflex function in rats. *Am J Physiol Regul Integr Comp Physiol.* 2010;298(1):R183–90. 10.1152/ajpregu.00176.2009 19907006

[38] Wu KLH Chan SHH Chan JY: Neuroinflammation and oxidative stress in rostral ventrolateral medulla contribute to neurogenic hypertension induced by systemic inflammation. *J Neuroinflammation.* 2012;9:212. 10.1186/1742-2094-9-212 22958438 PMC3462714

[39] Buggy J Fink GD Johnson AK: Prevention of the development of renal hypertension by anteroventral third ventricular tissue lesions. *Circ Res.* 1977;40(5 Suppl 1):I110–7. 858173

[40] Marvar PJ Thabet SR Guzik TJ: Central and peripheral mechanisms of T-lymphocyte activation and vascular inflammation produced by angiotensin II-induced hypertension. *Circ Res.* 2010;107(2):263–70. 10.1161/CIRCRESAHA.110.217299 20558826 PMC2921936

[41] Desson SE Ferguson AV: Interleukin 1beta modulates rat subfornical organ neurons as a result of activation of a non-selective cationic conductance. *J Physiol.* 2003;550(Pt 1):113–22. 10.1113/jphysiol.2003.041210 12879863 PMC2343005

[42] Wei SG Yu Y Zhang ZH: Proinflammatory cytokines upregulate sympathoexcitatory mechanisms in the subfornical organ of the rat. *Hypertension.* 2015;65(5):1126–33. 10.1161/HYPERTENSIONAHA.114.05112 25776070 PMC4393369

[43] Sriramula S Cardinale JP Lazartigues E: ACE2 overexpression in the paraventricular nucleus attenuates angiotensin II-induced hypertension. *Cardiovasc Res.* 2011;92(3):401–8. 10.1093/cvr/cvr242 21952934 PMC3286198

[44] Yu XJ Zhang DM Jia LL: Inhibition of NF-κB activity in the hypothalamic paraventricular nucleus attenuates hypertension and cardiac hypertrophy by modulating cytokines and attenuating oxidative stress. *Toxicol Appl Pharmacol.* 2015;284(3):315–22. 10.1016/j.taap.2015.02.023 25759242

[45] Zhang M Qin DN Suo YP: Endogenous hydrogen peroxide in the hypothalamic paraventricular nucleus regulates neurohormonal excitation in high salt-induced hypertension. *Toxicol Lett.* 2015;235(3):206–15. 10.1016/j.toxlet.2015.04.008 25891026

[46] Cutler JA Sorlie PD Wolz M: Trends in hypertension prevalence, awareness, treatment, and control rates in United States adults between 1988–1994 and 1999–2004. *Hypertension.* 2008;52(5):818–27. 10.1161/HYPERTENSIONAHA.108.113357 18852389

[47] Hart EC Charkoudian N: Sympathetic neural regulation of blood pressure: influences of sex and aging. *Physiology (Bethesda).* 2014;29(1):8–15. 10.1152/physiol.00031.2013 24382867

[48] Iams SG Wexler BC: Inhibition of the development of spontaneous hypertension in SH rats by gonadectomy or estradiol. *J Lab Clin Med.* 1979;94(4):608–16. 225399

[49] Sasaki T Ohno Y Otsuka K: Oestrogen attenuates the increases in blood pressure and platelet aggregation in ovariectomized and salt-loaded Dahl salt-sensitive rats. *J Hypertens.* 2000;18(7):911–7. 10.1097/00004872-200018070-00013 10930189

[50] Fogari R Preti P Zoppi A: Serum testosterone levels and arterial blood pressure in the elderly. *Hypertens Res.* 2005;28(8):625–30. 10.1291/hypres.28.625 16392765

[51] Svartberg J von Mühlen D Schirmer H: Association of endogenous testosterone with blood pressure and left ventricular mass in men. The Tromsø Study. *Eur J Endocrinol.* 2004;150(1):65–71. 10.1530/eje.0.1500065 14713281

[52] Bhattacharya RK Khera M Blick G: Effect of 12 months of testosterone replacement therapy on metabolic syndrome components in hypogonadal men: data from the Testim Registry in the US (TRiUS). *BMC Endocr Disord.* 2011;11:18. 10.1186/1472-6823-11-18 22044661 PMC3217857

[53] Li JY Zhu JC Dou JT: Effects of androgen supplementation therapy on partial androgen deficiency in the aging male: a preliminary study. *Aging Male.* 2002;5(1):47–51. 10.1080/tam.5.1.47.51 12040975

[54] Dahl LK Knudsen KD Ohanian EV: Role of the gonads in hypertension-prone rats. *J Exp Med.* 1975;142(3):748–59. 1165474 10.1084/jem.142.3.748 PMC2189927

[55] Ouchi Y Share L Crofton JT: Sex difference in the development of deoxycorticosterone-salt hypertension in the rat. *Hypertension.* 1987;9(2):172–7. 10.1161/01.HYP.9.2.172 3818014

[56] Chen YF Naftilan AJ Oparil S: Androgen-dependent angiotensinogen and renin messenger RNA expression in hypertensive rats. *Hypertension.* 1992;19(5):456–63. 10.1161/01.HYP.19.5.456 1568764

[57] Laflamme N Nappi RE Drolet G: Expression and neuropeptidergic characterization of estrogen receptors (ERalpha and ERbeta) throughout the rat brain: anatomical evidence of distinct roles of each subtype. *J Neurobiol.* 1998;36(3):357–78. 10.1002/(SICI)1097-4695(19980905)36:3<357::AID-NEU5>3.0.CO;2-V 9733072

[58] Rosas-Arellano MP Solano-Flores LP Ciriello J: Co-localization of estrogen and angiotensin receptors within subfornical organ neurons. *Brain Res.* 1999;837(1–2):254–62. 10.1016/S0006-8993(99)01672-8 10434010

[59] Simerly RB Chang C Muramatsu M: Distribution of androgen and estrogen receptor mRNA-containing cells in the rat brain: an *in situ* hybridization study. *J Comp Neurol.* 1990;294(1):76–95. 10.1002/cne.902940107 2324335

[60] Somponpun SJ Johnson AK Beltz T: Estrogen receptor-alpha expression in osmosensitive elements of the lamina terminalis: regulation by hypertonicity. *Am J Physiol Regul Integr Comp Physiol.* 2004;287(3):R661–9. 10.1152/ajpregu.00136.2004 15142833

[61] Spary EJ Maqbool A Batten TF: Oestrogen receptors in the central nervous system and evidence for their role in the control of cardiovascular function. *J Chem Neuroanat.* 2009;38(3):185–96. 10.1016/j.jchemneu.2009.05.008 19505570

[62] Xue B Zhao Y Johnson AK: Central estrogen inhibition of angiotensin II-induced hypertension in male mice and the role of reactive oxygen species. *Am J Physiol Heart Circ Physiol.* 2008;295(3):H1025–H1032. 10.1152/ajpheart.00021.2008 18599599 PMC2544478

[63] Xue B Badaue-Passos D Jr Guo F: Sex differences and central protective effect of 17beta-estradiol in the development of aldosterone/NaCl-induced hypertension. *Am J Physiol Heart Circ Physiol.* 2009;296(5):H1577–85. 10.1152/ajpheart.01255.2008 19270192 PMC2685359

[64] Xue B Zhang Z Beltz TG: Estrogen receptor-β in the paraventricular nucleus and rostroventrolateral medulla plays an essential protective role in aldosterone/salt-induced hypertension in female rats. *Hypertension.* 2013;61(6):1255–62. 10.1161/HYPERTENSIONAHA.111.00903 23608653 PMC3893074

[65] Li Z Hay M: 17-beta-estradiol modulation of area postrema potassium currents. *J Neurophysiol.* 2000;84(3):1385–91. 10980011 10.1152/jn.2000.84.3.1385

[66] Ciriello J Roder S: 17β-Estradiol alters the response of subfornical organ neurons that project to supraoptic nucleus to plasma angiotensin II and hypernatremia. *Brain Res.* 2013;1526:54–64. 10.1016/j.brainres.2013.06.038 23830850

[67] Pamidimukkala J Hay M: 17 beta-Estradiol inhibits angiotensin II activation of area postrema neurons. *Am J Physiol Heart Circ Physiol.* 2003;285(4):H1515–20. 10.1152/ajpheart.00174.2003 12829428

[68] Xue B Zhang Z Beltz TG: Genetic knockdown of estrogen receptor-alpha in the subfornical organ augments ANG II-induced hypertension in female mice. *Am J Physiol Regul Integr Comp Physiol.* 2015;308(6):R507–16. 10.1152/ajpregu.00406.2014 25552661 PMC4360069

[69] Gingerich S Krukoff TL: Estrogen in the paraventricular nucleus attenuates L-glutamate-induced increases in mean arterial pressure through estrogen receptor beta and NO. *Hypertension.* 2006;48(6):1130–6. 10.1161/01.HYP.0000248754.67128.ff 17075034

[70] Shih CD: Activation of estrogen receptor beta-dependent nitric oxide signaling mediates the hypotensive effects of estrogen in the rostral ventrolateral medulla of anesthetized rats. *J Biomed Sci.* 2009;16:60. 10.1186/1423-0127-16-60 19583861 PMC2717931

[71] St-Louis J Massicotte G: Chronic decrease of blood pressure by rat relaxin in spontaneously hypertensive rats. *Life Sci.* 1985;37(14):1351–7. 10.1016/0024-3205(85)90251-6 4046737

[72] Ma S Shen P Burazin TC: Comparative localization of leucine-rich repeat-containing G-protein-coupled receptor-7 (RXFP1) mRNA and [33P]-relaxin binding sites in rat brain: restricted somatic co-expression a clue to relaxin action? *Neuroscience.* 2006;141(1):329–44. 10.1016/j.neuroscience.2006.03.076 16725278

[73] Mumford AD Parry LJ Summerlee AJ: Lesion of the subfornical organ affects the haemotensive response to centrally administered relaxin in anaesthetized rats. *J Endocrinol.* 1989;122(3):747–55. 2809482 10.1677/joe.0.1220747

[74] Sun HJ Chen D Han Y: Relaxin in paraventricular nucleus contributes to sympathetic overdrive and hypertension via PI3K-Akt pathway. *Neuropharmacology.* 2016;103:247–56. 10.1016/j.neuropharm.2015.12.023 26746861

[75] Stumpe KO Kolloch R Higuchi M: Hyperprolactinaemia and antihypertensive effect of bromocriptine in essential hypertension. Identification of abnormal central dopamine control. *Lancet.* 1977;2(8031):211–4. 10.1016/S0140-6736(77)92832-X 69827

[76] Leaños-Miranda A Márquez-Acosta J Cárdenas-Mondragón GM: Urinary prolactin as a reliable marker for preeclampsia, its severity, and the occurrence of adverse pregnancy outcomes. *J Clin Endocrinol Metab.* 2008;93(7):2492–9. 10.1210/jc.2008-0305 18460570

[77] Oney T Bellmann O Kaulhausen H: Relationship between serum prolactin concentration, vascular angiotensin sensitivity and arterial blood pressure during third trimester pregnancy. *Arch Gynecol Obstet.* 1988;243(2):83–90. 10.1007/BF00932973 3401043

[78] Zhang L Curhan GC Forman JP: Plasma prolactin level and risk of incident hypertension in postmenopausal women. *J Hypertens.* 2010;28(7):1400–5. 10.1097/HJH.0b013e328339f254 20453663 PMC3139424

[79] Georgiopoulos GA Stamatelopoulos KS Lambrinoudaki I: Prolactin and preclinical atherosclerosis in menopausal women with cardiovascular risk factors. *Hypertension.* 2009;54(1):98–105. 10.1161/HYPERTENSIONAHA.109.132100 19451414

[80] Bole-Feysot C Goffin V Edery M: Prolactin (PRL) and its receptor: actions, signal transduction pathways and phenotypes observed in PRL receptor knockout mice. *Endocr Rev.* 1998;19(3):225–68. 10.1210/edrv.19.3.0334 9626554

[81] Patil MJ Henry MA Akopian AN: Prolactin receptor in regulation of neuronal excitability and channels. *Channels (Austin).* 2014;8(3):193–202. 10.4161/chan.28946 24758841 PMC4203747

[82] Brown RS Kokay IC Herbison AE: Distribution of prolactin-responsive neurons in the mouse forebrain. *J Comp Neurol.* 2010;518(1):92–102. 10.1002/cne.22208 19882722

[83] Hindmarch C Fry M Yao ST: Microarray analysis of the transcriptome of the subfornical organ in the rat: regulation by fluid and food deprivation. *Am J Physiol Regul Integr Comp Physiol.* 2008;295(6):R1914–20. 10.1152/ajpregu.90560.2008 18832082

[84] Durante W Johnson FK Johnson RA: Role of carbon monoxide in cardiovascular function. *J Cell Mol Med.* 2006;10(3):672–86. 10.1111/j.1582-4934.2006.tb00427.x 16989727 PMC3933149

[85] Zhao Y Vanhoutte PM Leung SW: Vascular nitric oxide: Beyond eNOS. *J Pharmacol Sci.* 2015;129(2):83–94. 10.1016/j.jphs.2015.09.002 26499181

[86] Abe K Kimura H: The possible role of hydrogen sulfide as an endogenous neuromodulator. *J Neurosci.* 1996;16(3):1066–71. 8558235 10.1523/JNEUROSCI.16-03-01066.1996 PMC6578817

[87] Hosoki R Matsuki N Kimura H: The possible role of hydrogen sulfide as an endogenous smooth muscle relaxant in synergy with nitric oxide. *Biochem Biophys Res Commun.* 1997;237(3):527–31. 10.1006/bbrc.1997.6878 9299397

[88] Kuo SM Lea TC Stipanuk MH: Developmental pattern, tissue distribution, and subcellular distribution of cysteine: alpha-ketoglutarate aminotransferase and 3-mercaptopyruvate sulfurtransferase activities in the rat. *Biol Neonate.* 1983;43(1–2):23–32. 6573924 10.1159/000241634

[89] Benavides GA Squadrito GL Mills RW: Hydrogen sulfide mediates the vasoactivity of garlic. *Proc Natl Acad Sci U S A.* 2007;104(46):17977–82. 10.1073/pnas.0705710104 17951430 PMC2084282

[90] Sun L Sun S Li Y: Potential biomarkers predicting risk of pulmonary hypertension in congenital heart disease: the role of homocysteine and hydrogen sulfide. *Chin Med J (Engl).* 2014;127(5):893–9. 24571884

[91] Sun NL Xi Y Yang SN: [Plasma hydrogen sulfide and homocysteine levels in hypertensive patients with different blood pressure levels and complications]. *Zhonghua Xin Xue Guan Bing Za Zhi.* 2007;35(12):1145–8. 18341820

[92] Wang C Han J Xiao L: Role of hydrogen sulfide in portal hypertension and esophagogastric junction vascular disease. *World J Gastroenterol.* 2014;20(4):1079–87. 10.3748/wjg.v20.i4.1079 24574782 PMC3921533

[93] Holwerda KM Bos EM Rajakumar A: Hydrogen sulfide producing enzymes in pregnancy and preeclampsia. *Placenta.* 2012;33(6):518–21. 10.1016/j.placenta.2012.02.014 22391326

[94] Wang K Ahmad S Cai M: Dysregulation of hydrogen sulfide producing enzyme cystathionine γ-lyase contributes to maternal hypertension and placental abnormalities in preeclampsia. *Circulation.* 2013;127(25):2514–22. 10.1161/CIRCULATIONAHA.113.001631 23704251

[95] d'Emmanuele di Villa Bianca R Sorrentino R Coletta C: Hydrogen sulfide-induced dual vascular effect involves arachidonic acid cascade in rat mesenteric arterial bed. *J Pharmacol Exp Ther.* 2011;337(1):59–64. 10.1124/jpet.110.176016 21228064

[96] Baragatti B Ciofini E Sodini D: Hydrogen sulfide in the mouse ductus arteriosus: a naturally occurring relaxant with potential EDHF function. *Am J Physiol Heart Circ Physiol.* 2013;304(7):H927–34. 10.1152/ajpheart.00718.2012 23376828

[97] Martelli A Testai L Breschi MC: Vasorelaxation by hydrogen sulphide involves activation of K _v_7 potassium channels. *Pharmacol Res.* 2013;70(1):27–34. 10.1016/j.phrs.2012.12.005 23287425

[98] Ariyaratnam P Loubani M Morice AH: Hydrogen sulphide vasodilates human pulmonary arteries: a possible role in pulmonary hypertension? *Microvasc Res.* 2013;90:135–7. 10.1016/j.mvr.2013.09.002 24035755

[99] Jackson-Weaver O Osmond JM Riddle MA: Hydrogen sulfide dilates rat mesenteric arteries by activating endothelial large-conductance Ca²⁺-activated K⁺ channels and smooth muscle Ca²⁺ sparks. *Am J Physiol Heart Circ Physiol.* 2013;304(11):H1446–54. 10.1152/ajpheart.00506.2012 23525712 PMC4073893

[100] Zhao W Zhang J Lu Y: The vasorelaxant effect of H _2_S as a novel endogenous gaseous K _ATP_ channel opener. *EMBO J.* 2001;20(21):6008–16. 10.1093/emboj/20.21.6008 11689441 PMC125693

[101] Ahmad FU Sattar MA Rathore HA: Exogenous hydrogen sulfide (H _2_S) reduces blood pressure and prevents the progression of diabetic nephropathy in spontaneously hypertensive rats. *Ren Fail.* 2012;34(2):203–10. 10.3109/0886022X.2011.643365 22229751

[102] Ahmad FU Sattar MA Rathore HA: Hydrogen sulphide and tempol treatments improve the blood pressure and renal excretory responses in spontaneously hypertensive rats. *Ren Fail.* 2014;36(4):598–605. 10.3109/0886022X.2014.882218 24502512

[103] Li L Whiteman M Guan YY: Characterization of a novel, water-soluble hydrogen sulfide-releasing molecule (GYY4137): new insights into the biology of hydrogen sulfide. *Circulation.* 2008;117(18):2351–60. 10.1161/CIRCULATIONAHA.107.753467 18443240

[104] Yan H Du J Tang C: The possible role of hydrogen sulfide on the pathogenesis of spontaneous hypertension in rats. *Biochem Biophys Res Commun.* 2004;313(1):22–7. 10.1016/j.bbrc.2003.11.081 14672692

[105] Yang G Wu L Jiang B: H _2_S as a physiologic vasorelaxant: hypertension in mice with deletion of cystathionine gamma-lyase. *Science.* 2008;322(5901):587–90. 10.1126/science.1162667 18948540 PMC2749494

[106] Ren YS Wu SY Wang XJ: Multiple hemodynamic effects of endogenous hydrogen sulfide on central nervous system in rats. *Chin Med J (Engl).* 2011;124(21):3468–75. 10.3760/cma.j.issn.0366-6999.2011.21.006 22340160

[107] Guo Q Jin S Wang XL: Hydrogen sulfide in the rostral ventrolateral medulla inhibits sympathetic vasomotor tone through ATP-sensitive K ^+^ channels. *J Pharmacol Exp Ther.* 2011;338(2):458–65. 10.1124/jpet.111.180711 21558438

[108] Gan XB Liu TY Xiong XQ: Hydrogen sulfide in paraventricular nucleus enhances sympathetic activity and cardiac sympathetic afferent reflex in chronic heart failure rats. *PLoS One.* 2012;7(11):e50102. 10.1371/journal.pone.0050102 23166827 PMC3499499

[109] Kuksis M Smith PM Ferguson AV: Hydrogen sulfide regulates cardiovascular function by influencing the excitability of subfornical organ neurons. *PLoS One.* 2014;9(8):e105772. 10.1371/journal.pone.0105772 25144759 PMC4140834

[110] Malik R Ferguson AV: Hydrogen sulfide depolarizes neurons in the nucleus of the solitary tract of the rat. *Brain Res.* 2016;1633:1–9. 10.1016/j.brainres.2015.12.029 26721687

[111] Khademullah CS Ferguson AV: Depolarizing actions of hydrogen sulfide on hypothalamic paraventricular nucleus neurons. *PLoS One.* 2013;8(5):e64495. 10.1371/journal.pone.0064495 23691233 PMC3656899

[112] Rodrigo R Libuy M Feliú F: Oxidative stress-related biomarkers in essential hypertension and ischemia-reperfusion myocardial damage. *Dis Markers.* 2013;35(6):773–90. 10.1155/2013/974358 24347798 PMC3856219

[113] Rodrigo R Prat H Passalacqua W: Relationship between oxidative stress and essential hypertension. *Hypertens Res.* 2007;30(12):1159–67. 10.1291/hypres.30.1159 18344620

[114] Cowley AW Jr Yang C Zheleznova NN: Evidence of the Importance of Nox4 in Production of Hypertension in Dahl Salt-Sensitive Rats. *Hypertension.* 2016;67(2):440–50. 10.1161/HYPERTENSIONAHA.115.06280 26644237 PMC4713301

[115] Dikalova A Clempus R Lassègue B: Nox1 overexpression potentiates angiotensin II-induced hypertension and vascular smooth muscle hypertrophy in transgenic mice. *Circulation.* 2005;112(17):2668–76. 10.1161/CIRCULATIONAHA.105.538934 16230485

[116] Braga VA Colombari E Jovita MG: Angiotensin II-derived reactive oxygen species underpinning the processing of the cardiovascular reflexes in the medulla oblongata. *Neurosci Bull.* 2011;27(4):269–74. 10.1007/s12264-011-1529-z 21788998 PMC5560308

[117] Braga VA Medeiros IA Ribeiro TP: Angiotensin-II-induced reactive oxygen species along the SFO-PVN-RVLM pathway: implications in neurogenic hypertension. *Braz J Med Biol Res.* 2011;44(9):871–6. 10.1590/S0100-879X2011007500088 21755262

[118] Kishi T Hirooka Y Kimura Y: Increased reactive oxygen species in rostral ventrolateral medulla contribute to neural mechanisms of hypertension in stroke-prone spontaneously hypertensive rats. *Circulation.* 2004;109(19):2357–62. 10.1161/01.CIR.0000128695.49900.12 15117836

[119] Zimmerman MC Lazartigues E Lang JA: Superoxide mediates the actions of angiotensin II in the central nervous system. *Circ Res.* 2002;91(11):1038–45. 10.1161/01.RES.0000043501.47934.FA 12456490

[120] Lob HE Marvar PJ Guzik TJ: Induction of hypertension and peripheral inflammation by reduction of extracellular superoxide dismutase in the central nervous system. *Hypertension.* 2010;55(2):277–83, 6p following 283. 10.1161/HYPERTENSIONAHA.109.142646 20008675 PMC2813894

[121] Halaas JL Gajiwala KS Maffei M: Weight-reducing effects of the plasma protein encoded by the obese gene. *Science.* 1995;269(5223):543–6. 10.1126/science.7624777 7624777

[122] Carmichael CY Wainford RD: Hypothalamic signaling mechanisms in hypertension. *Curr Hypertens Rep.* 2015;17(5):39. 10.1007/s11906-015-0550-4 25860531 PMC4392165

[123] Stump M Mukohda M Hu C: PPARγ Regulation in Hypertension and Metabolic Syndrome. *Curr Hypertens Rep.* 2015;17(12):89. 10.1007/s11906-015-0601-x 26462805 PMC6766749

